# Building Health System Capacity through Medical Education: A Targeted Needs Assessment to Guide Development of a Structured Internal Medicine Curriculum for Medical Interns in Botswana

**DOI:** 10.29024/aogh.22

**Published:** 2018-04-30

**Authors:** Michael J. Peluso, Neo Tapela, John Langeveldt, Margaret E. Williams, Kagiso Mochankana, Kebonye Motseosi, Brian Ricci, Adam Rodman, Cecil Haverkamp, Miriam Haverkamp, Rosa Maoto, Rebecca Luckett, Detlef Prozesky, Oathokwa Nkomazana, Tomer Barak

**Affiliations:** 1 Botswana-Harvard Partnership, Gaborone, Botswana, ZA; 2 Department of Medicine and Division of Global Health Equity, Brigham and Women’s Hospital, Boston, MA, US; 3 Harvard Medical School, Boston, MA, US; 4 Botswana Ministry of Health, Gaborone, Botswana, ZA; 5 Bamalete Lutheran Hospital, Ramotswa, Botswana, ZA; 6 Department of Medicine, Scottish Livingstone Hospital, Molepolole, Botswana, ZA; 7 Department of Medicine, Beth Israel Deaconess Medical Center, Boston, US; 8 The Ohio State University Wexner Medical Center, Columbus, OH, US; 9 Medical Internship Training Programme, Gaborone, Botswana, ZA; 10 Department of Medicine, Oregon Health and Science University, Portland, OR, US; 11 University Research Co., Gaborone, Botswana, ZA; 12 Department of Medicine, Princess Marina Hospital, Gaborone, Botswana, ZA; 13 Botswana-UPenn Partnership, Gaborone, Botswana, ZA; 14 Department of Obstetrics and Gynecology, Scottish Livingstone Hospital, Molepolole, Botswana, ZA; 15 Department of Obstetrics and Gynecology, Beth Israel Deaconess Medical Center, Boston, MA, US; 16 Department of Medical Education, University of Botswana, Gaborone, Botswana, ZA; 17 Faculty of Medicine, University of Botswana, Gaborone, Botswana, ZA

## Abstract

**Background:**

Medical internship is the final year of training before independent practice for most doctors in Botswana. Internship training in Botswana faces challenges including variability in participants’ level of knowledge and skill related to their completion of medical school in a variety of settings (both foreign and domestic), lack of planned curricular content, and limited time for structured educational activities. Data on trainees’ opinions regarding the content and delivery of graduate medical education in settings like Botswana are limited, which makes it difficult to revise programs in a learner-centered way.

**Objective:**

To understand the perceptions and experiences of a group of medical interns in Botswana, in order to inform a large curriculum initiative.

**Methods:**

We conducted a targeted needs assessment using structured interviews at one district hospital. The interview script included demographic, quantitative, and free- response questions. Fourteen interns were asked their opinions about the content and format of structured educational activities, and provided feedback on the preferred characteristics of a new curriculum. Descriptive statistics were calculated.

**Findings:**

In the current curriculum, training workshops were the highest-scored teaching format, although most interns preferred lectures overall. Specialists were rated as the most useful teachers, and other interns and medical officers were rated as average. Interns felt they had adequate exposure to content such as HIV and tuberculosis, but inadequate exposure to areas including medical emergencies, non-communicable diseases, pain management, procedural skills, X-ray and EKG interpretation, disclosing medical information, and identifying career goals. For the new curriculum, interns preferred a structured case discussion format, and a focus on clinical reasoning and procedural skills.

**Conclusions:**

This needs assessment identified several foci for development, including a shift toward interactive sessions focused on skill development, the need to empower interns and medical officers to improve teaching skills, and the value of shifting curricular content to mirror the epidemiologic transition occurring in Botswana. Interns’ input is being used to initiate a large curriculum intervention that will be piloted and scaled nationally over the next several years. Our results underscore the value of seeking the opinion of trainees, both to aid educators in building programs that serve them and in empowering them to direct their education toward their needs and goals.

## Background

Ensuring the quality of medical training is an important requirement for long-term retention of doctors and the delivery of quality healthcare [1,2,3,4]. While medical internship training has in some settings become an intermediate step towards higher-level qualifications, in many settings it remains a concluding step in training for most individuals before medical licensure and independent practice.

In Botswana, a middle-income country of approximately two million inhabitants in southern Africa, only a small proportion of doctors are trained as specialists and the bulk of medical care is provided by general practitioners (referred to as “medical officers”) who have undergone one year of post-medical school internship training before beginning independent practice [3]. Despite gains made in the fight against HIV/AIDS, the medical system in Botswana face challenges that include a shortage of doctors, resource constraints, and widespread epidemics of HIV and tuberculosis in addition to a growing burden of non-communicable diseases [2,5,6,7,8,9]. Internship training in Botswana targets doctors from diverse medical school backgrounds (in-country and foreign); takes place in a variety of clinical settings which differ in available resources and expertise of trainers (specialists vs. non-specialized medical officers); and is intended, over a brief period of time, to produce doctors competent to practice independently across a wide scope of medical, pediatric, surgical, and obstetric areas. In addition, internship training in Botswana seeks to contribute to the wider national effort of promoting the retention of medical doctors [1,3,4].

Botswana’s revised national Medical Internship Training Programme (MIT) was launched in 2014 in an effort to increase the number and quality of doctors serving in Botswana’s public health system, particularly in remote and rural areas. The MIT aims to optimize the transition of recently graduated medical doctors to their roles as fully autonomous medical practitioners. Internship training, under MIT, takes place at five district hospitals and two referral centers over one year and consists of 3-month rotations in internal medicine, pediatrics, obstetrics and gynecology, and general surgery. Following internship training, graduates are assigned to practice as medical officers in a variety of settings. Internship training is thus a critical step in the transition from supervised medical training to independent practice. To guide this transition, the Botswana Health Professions Council (BHPC) has put forth competencies expected of medical doctors and the MIT has developed an intern handbook to guide supervisors and trainees during internship.

In addition to clinical apprenticeship, BHPC emphasizes the need for structured learning activities at each training site. These activities are defined as department- and hospital-level educational sessions that offer opportunities for structured learning and continuing professional development; they are, scheduled at a regular time and place, and attendance is supported by supervisors and expected of trainees. The format and content of structured learning activities are developed separately at each training site and are thus highly variable. The lack of a formalized, longitudinal curriculum of structured learning activities likely leads to duplication of work by internship supervisors, as well as variability in training outcomes and graduates’ preparedness for future clinical assignments.

To address some of these challenges, the Botswana Ministry of Health, University of Botswana Faculty of Medicine, MIT, and academic partners undertook the design of a comprehensive national curriculum of structured learning activities for MIT trainees. Here, we describe the findings of a targeted needs assessment of learners and the internship learning environment at one district hospital, conducted to inform this project.

## Methods

We conducted one-on-one structured interviews with fourteen medical interns over the course of one week in October 2015. Eligible participants included all fourteen medical interns training at one district hospital (Scottish Livingstone Hospital) at the time. Participation was voluntary, and interns provided written informed consent for their participation.

Interviews included demographic, quantitative, and free-response questions regarding the format and content of the interns’ educational experience. The content of the interviews and composition of the questions were determined in committee between three of the authors (MJP, NT, TB) with experience in working with medical interns in Botswana. Interviews were conducted in English by one investigator (MJP) who had experience working at the site, but was not responsible for evaluating the interns in any capacity.

Responses were recorded anonymously on paper and later input into a password-protected electronic database.

The survey section on curricular format evaluated five types of structured learning activities, as defined in Table 1. Interns rated each activity on a 5-point Likert scale according to the degree to which the activity facilitated information retention, effective learning, and improved patient management skills. An aggregate score for each activity was calculated by averaging these three sub-scores. Interns were also asked to rank the formats in order of overall utility. Similarly, interns were asked to rank the various types of instructors in the curriculum regarding their effectiveness as teachers.

**Table 1:** Session format descriptions for structured learning activities.

Session Type	Description

Current Structured Learning Activity Formats	
Lecture	any formal lecture given by a senior clinician or educator
Intern report	a case presentation and discussion led by an intern with or without facilitation from a senior clinician or educator
Journal club	a session devoted to critical appraisal of a study published in the scientific literature
Training workshop	one of several externally developed training sessions in which large cohorts of trainees participate, for example HIV provider training or Advanced Cardiac Life Support training
Morbidity and Mortality conference	a multi-disciplinary conference in which patient cases resulting in adverse outcomes are discussed for education and quality improvement purposes

Proposed Structured Learning Activity Formats	
Flipped classroom	interns receive materials beforehand, complete work at home, and then have a follow-up discussion in a group setting
Structured case discussion	a session focused on an approach to a symptom, physical finding, laboratory result, or imaging study, embedded within a patient case
Self-directed learning	a topic that is assigned for independent study
Unstructured	discussion with a senior clinician about a topic or topics that are not
session	preplanned, but brought up by the interns during that session

For the assessment of curriculum content, interns characterized their exposure to topics in their training as “too much,” “just right,” or “too little” and additionally assigned importance to each topic using a 5-point Likert scale. We predefined a threshold for significance as more than 50% of respondents identifying “too little” exposure to a topic *and* that topic having an average importance rating of 4 or greater. Domain scores for four domains (knowledge, skills, communication, professionalism) were calculated by averaging all topic scores within that domain. Interns were asked to select which domain should be the focus of future curriculum development.

Questions regarding curriculum development asked interns to state their ideal duration of a structured learning activity in minutes, their ideal class size, and the number of sessions they felt they should have per week. They were asked to rate four potential new educational formats on a 5-point scale, and to provide open-ended commentary on each suggested format. These formats are reviewed in Table 1.

Interns were also asked if they preferred a symptom-based or system-based curriculum, defined as focused on evaluation and management of a patient’s presenting signs or symptoms versus focused on a disease- or organ-system approach to curricular content, respectively.

Data analyses involved calculation of basic descriptive statistics. All analyses were conducted using SPSS version 24.0 (IBM Corp, Armonk, New York) and GraphPad Prism version 5.0d (GraphPad Software, San Diego, California). The data were initially collected for quality improvement; analysis for research purposes received ethical approval from the Botswana Health Resources Development Council and the Beth Israel Deaconess Medical Center Committee on Clinical Investigations.

## Results

### Demographic characteristics of participants

All fourteen eligible interns at the targeted facility elected to participate. The demographics of respondents are described in Table 2. Broken down, 6 of 14 (43%) were male, 9 of 14 (64%) were 20 to 25 years old, and 8 of 14 (57%) had graduated from medical school at the University of Botswana, while 6 of 14 (43%) had graduated from a medical school outside of Botswana. Five participants had completed the full internship year, and nine had completed a portion of the internship year.

**Table 2:** Demographic characteristics of survey respondents.

Category	n (%)

Gender	
Male	6 (43%)
Female	8 (57%)

Age	
20–25	9 (64%)
26–30	5 (36%)

Medical School	
University of Botswana	8 (57%)
Outside Botswana	6 (43%)

### Preferences in the current curriculum

Table 3 displays intern preferences regarding educational sessions. Interns felt that training workshops best prepared them to manage patients, retain information, and learn effectively. This was reflected in the aggregate scores, in which training workshops scored highest, followed by intern reports, morbidity and mortality (M&M) conferences, lectures, and journal clubs. However, when asked to rank these sessions in order of overall utility, interns rated lectures the highest, followed by intern reports, training workshops, journal clubs, and M&M conferences.

**Table 3:** Opinions on current educational formats and teachers.

	Manage Patients	Retain Information	Learn Effectively	Aggregate Score	No. ranking “most useful”

Format					
Lectures	4.07 (0.83)	3.14 (0.66)	4.00 (0.68)	3.73 (0.83)	5 (36%)
Intern report	3.79 (0.89)	3.79 (0.80)	4.21 (0.80)	3.93 (0.84)	4 (29%)
Training workshops	4.43 (0.79)	4.29 (0.48)	4.71 (0.49)	4.47 (0.60)	3 (21%)
Journal club	3.14 (0.66)	3.29 (0.83)	3.43 (0.65)	3.29 (0.71)	1 (7%)
M&M	3.86 (0.77)	3.93 (0.83)	4.07 (0.83)	3.95 (0.79)	1 (7%)

Teachers					
Interns	–	–	–	–	1 (7%)
Medical Officers	–	–	–	–	0 (0%)
Residents	–	–	–	–	2 (14%)
Specialists	–	–	–	–	11 (79%)

Interns ranked specialists as the most useful teachers (11/14, 79%), followed by residents, other interns, and medical officers.

### Curriculum content

Table 4 describes interns’ impressions of the content of their internship curriculum. Within the knowledge domain, 12 of 14 interns (86%) felt that there was adequate exposure to principles of HIV and tuberculosis care. Interns identified significant deficiencies in the following areas; medical emergencies, noncommunicable diseases, pain management, preventive medicine, and cardiopulmonary resuscitation. In the skills domain, interns felt that they had adequate exposure to patient management and clinical decision-making, analyzing and interpreting scientific data (i.e, critically appraising journal articles), and management of fluid and electrolyte abnormalities. They identified deficiencies in being prepared to independently perform procedures (e.g., thoracentesis, lumbar puncture), independently interpret X-rays and ECGs, perform ultrasounds, manage medical emergencies, apply data from scientific studies to patient care, and manage insulin regimens. Interns felt that their exposure within the communication domain was adequate, with the exception of learning to disclose medical information in a sensitive manner. Within the professionalism domain, they identified recognition of individual limitations and strengths, handling medical errors, and identifying career goals as areas requiring more exposure. Overall domain scores demonstrated that interns perceived items in the skills domain as the most important, followed by the knowledge domain, professionalism domain, and communication domain.

**Table 4:** Opinion on the extent and importance of knowledge-based content in the current internship curriculum, October 2015.

Content Topic	Too Much n (%)	Just Enough n (%)	Too Little n (%)	Perceived Importance Mean (SD)

Knowledge Domain				3.99 (1.00)
HIV	2 (14%)	10 (71%)	2 (14%)	4.14 (0.86)
Tuberculosis	1 (7%)	12 (86%)	1 (7%)	4.14 (0.86)
Medical emergencies	0 (0%)	1 (7%)	13 (93%)	4.79 (0.43)
Non-communicable diseases	0 (0%)	3 (21%)	11 (79%)	4.07 (0.92)
Cancer	0 (0%)	0 (0%)	14 (100%)	3.5 (0.76)
Pain management	0 (0%)	4 (29%)	10 (71%)	4.07 (0.92)
Psychiatry	0 (0%)	0 (0%)	14 (100%)	3.5 (1.02)
End-of-life care	0 (0%)	0 (0%)	14 (100%)	3.14 (0.77)
Preventative medicine	0 (0%)	1 (7%)	13 (93%)	4.36 (1.2)
CPR	0 (0%)	3 (21%)	11 (79%)	4.86 (0.36)
Social determinants of health	0 (0%)	3 (21%)	11 (79%)	3.50 (1.22)
Outpatient medicine	0 (0%)	2 (14%)	12 (86%)	3.86 (1.1)

Skills Domain				4.04 (0.95)
Taking histories	1 (7%)	9 (64%)	4 (29%)	3.21 (1.1)
Doing physical examinations	0 (0%)	11 (79%)	3 (21%)	3.64 (1.0)
Patient management and clinical decision-making	0 (0%)	10 (71%)	4 (29%)	4.36 (0.75)
Doing procedures (e.g., thoracentesis, lumbar puncture)	0 (0%)	7 (50%)	7 (50%)	4.50 (0.76)
Evaluating laboratory data	0 (0%)	9 (64%)	5 (36%)	3.79 (0.80)
Interpreting X-rays	1 (7%)	5 (36%)	8 (57%)	4.14 (0.86)
Interpreting ECGs	0 (0%)	6 (43%)	8 (57%)	4.36 (0.84)
Performing and interpreting ultrasounds	0 (0%)	0 (0%)	14 (100%)	4.86 (0.36)
Dealing with emergencies	0 (0%)	5 (36%)	9 (64%)	4.79 (0.43)
Analyzing and interpreting scientific data (i.e., from a journal)	0 (0%)	8 (57%)	6 (43%)	4.07 (0.83)
Applying scientific data to patient care	0 (0%)	5 (36%)	9 (64%)	4.29 (0.83)
Managing stress	0 (0%)	1 (7%)	13 (93%)	3.93 (1.0)
Managing time	0 (0%)	3 (21%)	11 (79%)	3.64 (0.93)
Prioritizing tasks	0 (0%)	5 (36%)	9 (64%)	3.71 (0.91)
Recognizing cognitive biases in medicine	0 (0%)	2 (14%)	12 (86%)	4.21 (0.70)
Electrolyte management	0 (0%)	9 (64%)	5 (36%)	4.21 (0.89)
Fluid management	0 (0%)	11 (79%)	3 (21%)	4.21 (0.58)
DVT prophylaxis	0 (0%)	7 (50%)	7 (50%)	3.64 (1.22)
Bowel management	0 (0%)	9 (64%)	5 (36%)	3.07 (0.83)
Quality improvement skills	0 (0%)	4 (29%)	10 (71%)	3.64 (1.22)
Insulin management	0 (0%)	3 (21%)	11 (79%)	4.57 (0.65)

Communication Domain				3.71 (1.18)
Listening to patients	1 (7%)	7 (50%)	6 (43%)	3.57 (1.2)
Communicating with patients	0 (0%)	8 (57%)	6 (43%)	3.86 (1.2)
Assessing patients’ knowledge of their disease and prognosis	0 (0%)	5 (36%)	9 (64%)	3.86 (1.5)
Disclosing medical information in a sensitive manner	0 (0%)	8 (57%)	6 (43%)	4.00 (1.2)
Eliciting and responding to patients’ worries or fears	0 (0%)	8 (57%)	6 (43%)	3.7 (1.3)
Communicating with patients’ relatives	0 (0%)	10 (71%)	4 (29%)	3.5 (0.86)
Communicating with professional colleagues	1 (7%)	11 (79%)	2 (14%)	3.5 (1.1)

Professionalism Domain				3.92 (1.11)
Recognize your limitations and strengths	0 (0%)	5 (36%)	9 (64%)	4.14 (0.77)
Work in a team	0 (0%)	11 (79%)	3 (21%)	3.50 (1.16)
Adapt to different clinical environments	0 (0%)	12 (86%)	2 (14%)	3.36 (1.21)
Self-audit	1 (7%)	3 (21%)	10 (71%)	3.93 (1.21)
Admit to mistakes and talk about them to improve practice	0 (0%)	7 (50%)	7 (50%)	4.07 (1.27)
Identify career goals	0 (0%)	2 (14%)	12 (86%)	4.57 (0.65)

### Preferences for new curriculum development

Table 5 describes preferences for curriculum development. Interns reported that they felt the ideal duration for curriculum sessions was, on average, 47 minutes; the ideal number of education sessions per week was four; and fewer than 15 participants was ideal for their learning in educational sessions. They preferred morning sessions, citing higher attentiveness and the value of allowing post-call interns, who may not be present later in the day, to participate.

**Table 5:** Opinions on potential new formats for curriculum sessions.

	Preference mean (SD) unless otherwise specified

Potential new format	
Flipped classroom	3.43 (1.3)
Self-education modules	2.36 (1.2)
Structured case discussions/Case-based learning	4.71 (0.47)
Unstructured sessions (topic determined same day)	2.93 (1.4)

Ideal session characteristics	
Duration (minutes)	47 (11.7)
Number per week	4 (0.96)
Size of class (individuals)	14.7 (5.3)
Time of day – Morning	14 (100%)

System or symptom based curriculum	
Symptom-based	13 (93%)
System-based	1 (7%)

Preferred area of focus for new curriculum	
Knowledge	4 (29%)
Skills	9 (64%)
Communication	0 (0%)
Professional development	1 (7%)

The most popular new format was the structured case discussion. Interns were less interested in flipped classroom, self-education modules, or unstructured teaching sessions. Below are some representative comments offering insight into these preferences.

Regarding the flipped classroom model, representative responses included the following:

“People probably won’t review materials [distributed beforehand] but if you are responsible for a specific task with regard to the materials you will be more likely to do the work.”

“[There is] no time to review materials outside of the hospital.”

Regarding self-directed education materials, they said:

“[They] would be good because of personal growth and accountability, but the risk is that there are no consequences for not doing the work.”

Regarding case-based discussions, they said:

“[These sessions will] help fill in gaps in management”

“[These sessions will] add more to learning, and we will be able to apply what we learned.”

Regarding spontaneous, unstructured sessions, they said:

“At the end of the day, you need to be prepared. This [type of session] will not lead to in-depth discussions.”

The majority of interns preferred new curriculum sessions focus on skill development. Comments explaining this preference included the following:

“Medical school is about knowledge, internship is about skills and should prepare you for the skills you will need as a medical officer. Professional development will happen naturally.”

“Especially in the district, emphasis is on practical performance. You can have knowledge but if you can’t act on it, you are useless.”

Thirteen out of fourteen (93%) favored a symptom-based curriculum that focused on patients’ presenting complaints. Representative comments included the following:

“Patients come with symptoms, not with ‘I have a kidney problem.’”

“A symptoms approach doesn’t limit you – the patient can come in with a symptom that doesn’t apply to a specific system.”

“In medical school, you learn a disease- or system-based approach, but as an intern you develop approaches to patient presentations.”

## Discussion

This article reports the results of a needs assessment conducted through structured interviews with 14 medical interns at a district-level hospital in Botswana. The needs assessment was intended to inform the design and implementation of a formal national curriculum of structured learning activities that would enhance clinical education during internship training, standardize training experiences and outcomes across internship sites, and better prepare interns for independent practice. Interns’ responses reveal that the existing curriculum at the targeted institution may not be adequately meeting the perceived educational needs of our trainees and identified several areas of focus for future development of graduate medical education in this setting.

### Shifting content to mirror the epidemiologic transition

While interns felt that curricular content provided adequate exposure to areas that have traditionally been the focus of the healthcare system in Botswana and its regional neighbors (i.e., HIV and tuberculosis), the coverage of many of the noncommunicable diseases and their acute and chronic complications was felt to be inadequate. This is especially important as countries in the region begin to focus more resources on noncommunicable diseases and preventive care, and it suggests that efforts need to be undertaken on the part of educators to ensure that there is structured exposure to these conditions and their management. As the burden of these diseases grows, medical training programs must mirror the shift of emphasis that is already occurring in many public health systems.

### Optimizing content delivery

While training workshops achieved the highest overall aggregate scores, interns ranked lectures and intern reports as the most useful formats, suggesting that both receiving information and actively presenting it are of perceived benefit. The fact that M&M sessions were the least-preferred format despite having the second-highest aggregate score warrants further investigation as concepts such as reflective practice and quality improvement are further emphasized. We postulate that this discrepancy may result from challenges in establishing a safe and nonjudgmental environment for these often-sensitive sessions. The results also suggest that focus on case-based learning may be of benefit to trainees.

### Prioritization of skills training

Interns’ prioritization of skills training should be taken in the context of their perceptions of increased responsibility in their transition to the medical officer role and may reflect their need to rapidly acquire the ability to practice independently in anticipation of limitations on further formal training. Interns tended to deprioritize communication skills and professional development, and several suggested that these would happen organically with practice.

However, these are areas that are receiving more explicit focus in training programs throughout the world, particularly in high-income settings [10,11]. We postulate that the deprioritization of these domains is due to the high workload during internship and the impression of the need to master the basics before focusing on effective communication or personal career development. Communication skills in medical care have been demonstrated to be of great importance [12,13], and further efforts to explore these domains in sub-Saharan Africa should be explored.

### Building capacity through a focus on peer- and near-peer teaching

Interns considered specialists and residents (i.e., specialists-in-training) to be the most effective educational session leaders. This is particularly notable given that not all interns in similar settings have consistent access to specialist instructors. Interns rated their peers (other interns) as average teachers, suggesting that interventions aimed at improving their abilities as instructors may be beneficial. While nonspecialized medical officers were considered to be the least effective teachers, they are the individuals to which interns have the most exposure. Interventions focused on training and empowering nonspecialist medical officers as educators may be of value in building educational capacity in this setting.

### Future directions

Based on these results, we have set out to develop a curricular package of structured learning activities, beginning with the field of internal medicine. This curriculum takes into account interns’ preferences, including the implementation of protected time for structured educational sessions, which are scheduled four mornings per week. Furthermore, the curricular package deemphasizes traditional topic overviews in favor of case-based, clinical reasoning and skills-focused sessions that aim to deliver practical tools to allow interns to apply learned content in their day-to-day practice.

The curriculum package, which includes approximately 100 hours of content in internal medicine, is specifically designed to be implementable in a modular fashion across the variety of internship training sites in Botswana and by different levels of instructors (e.g., specialists, residents, medical officers, and interns) as per their availability at each site.

It is being initially piloted at one training site and, if successful, disseminated across MIT sites in Botswana. We plan to follow a similar procedure for the development of educational packages in obstetrics and gynecology, surgery, and pediatrics. Our hope is that by empowering instructors of different training levels to deliver content that is structured, up to date, practical, and relevant to the clinical practice setting, this project will contribute to the standardization of internship training across Botswana and the graduation of independent medical practitioners with a consistent level of knowledge, skills, and clinical competence.

### Limitations

This needs assessment is limited by its small sample size and participants, which is a convenience sample from one training site. Therefore, generalizations regarding the preferences of interns at other district hospitals or referral hospitals should be made with caution. While our sample was small, it was reflective of the overall population of medical interns in Botswana in terms of age and medical school background. Notes were transcribed but not audiorecorded by the interviewer, leading to the possibility of errors. The statistical analysis is meant to demonstrate trends in the data, but our sample was not robust for statistical comparisons to be made (for example, between interns who trained in-country and at foreign medical schools).

Finally, this project does not explicitly address the need to train teachers in this setting, which will be important for curriculum delivery. Despite these limitations, we believe that these findings are of value for the purposes of informing curriculum development and human resource capacity building in similar settings, as information available on graduate medical education in sub-Saharan Africa is extremely limited.

## Conclusion

This study provides useful preliminary data on the preferences and perceptions of medical interns at one district-level hospital in Botswana, which may be useful to educators working in similar settings. We have used these data to initiate development of a large-scale curriculum intervention that will be piloted at one facility and scaled nationally over the course of the next several years. Our results underscore the value of seeking the opinion and input of medical trainees, both to aid educators in building programs to serve them and in empowering trainees to direct their education toward their own needs and goals.

## Declarations

Ethics approval and consent to participate: Analysis for research purposes received ethical approval from the Botswana Health Resources Development Council. All participants signed a written consent to participate.

## Previous Presentations

An abstract related to this project will be presented at the Consortium of Universities for Global Health meeting in Washington, D.C. in April 2017.

## Availability of data and material

The data and material used in this study are available from the corresponding author on reasonable request.

## Abbreviations

MIT, Medical Internship Training Programme; BHPC, Botswana Health Professions Council; M&M, Morbidity and Mortality Conference.

## Author Inforamations

MJP is a global health equity resident and has been working intermittently in Botswana for two years as the coordinator of the internship curriculum project. NT serves as the head of the non-communicable disease program at the Botswana Ministry of Health and is a practicing physician in Botswana. JL, KM, and KM are graduates of the MIT currently working in the Botswana health system. MEW and AR are former and current global health fellows, a role involving clinical work and internship training at Scottish Livingstone Hospital. BR works on medical education and noncommunicable disease education at Scottish Livingstone Hospital. CH, DP, and ON were involved in the development of the MIT, and remain active in directing the program and in other medical education initiatives in Botswana. RM is the current program administrator. MH is a practicing physician and infectious disease specialist at Princess Marina Hospital, and is involved in internship education at that site. RL is a practicing obstetrician at Scottish Livingstone Hospital and is involved in internship training and education at that site; she is directing the development of an obstetric curriculum that will follow piloting of the internal medicine curriculum. TB is the internship site director at Scottish Livingstone Hospital, where he works as a physician, and is active in the further development of the MIT.
